# Prophylactic gabapentin during head and neck cancer therapy: a systematic review and meta-analysis

**DOI:** 10.1007/s00520-023-07683-w

**Published:** 2023-03-17

**Authors:** Loren E. Smith, Barbara A. Murphy, Derek K. Smith

**Affiliations:** 1 Department of Anesthesiology, Vanderbilt University Medical Center, Nashville, TN USA; 2 Department of Medicine, Vanderbilt University Medical Center, Nashville, TN USA; 3 American Dental Association Science and Research Institute, Chicago, IL USA

**Keywords:** Gabapentin, Opioids, Systematic review, Meta-analysis, Head and neck cancer

## Abstract

**Purpose:**

This review was designed to compile the currently available evidence on the prophylactic use of gabapentin in the head and neck cancer patient population.

**Methods:**

A systematic search was conducted of PubMed, Web of Science, and Google Scholar to identify articles related to the use of prophylactic gabapentin in patients undergoing head and neck cancer therapy. Candidate studies were screened for inclusion and a subsequent bias assessment was conducted by multiple reviewers. Meta-analysis was conducted in cases in which the studies used compatible outcome measures.

**Results:**

Ten studies were identified that met the inclusion criteria and were assessed for bias. Among the four small studies that examined pain prevention, 2 were positive and 2 were inconclusive. Three of the four studies examiniRDng opioid use noted less need for opioids in the treatment arm. Meta-analysis of the pertinent studies showed no difference in feeding tube placement (RD = 0.64%, 95%CI: (− 25.8%, 27.1%), *p* = 0.962) but substantially less weight loss among those in the treatment arm (*p* = 0.047).

**Conclusion:**

Prophylactic gabapentin appears to be a promising treatment option for preventing pain, reducing opioids, and reducing weight loss in patients undergoing head and neck cancer therapy. However, the studies on the treatment to date are small and several have a substantial risk of bias.

## Introduction

An estimated 65,000 patients will be diagnosed with head and neck cancer (HNC) in the USA each year resulting in over 15,000 deaths annually [1]. Unfortunately, the incidence of locally advanced HNC has been increasing particularly among African American and male subpopulations [2]. In addition, there has been a rise in human papillomavirus (HPV)-associated oropharyngeal cancers which is associated with both younger patients and improved survival [3]. This demographic shift brings supportive and survivorship care to the forefront. This is particularly salient in younger survivors who will have to contend with treatment-related morbidities for a greater amount of time.

Patients undergoing concurrent chemotherapy and radiation therapy for HNC experience a wide variety of acute toxicities secondary to treatment. One of the most challenging toxicities to manage is mucositis and associated pain. Mucositis-associated pain can lead to numerous complications including dysphagia, odynophagia, dehydration, and weight loss. This pain may lead to feeding tube placement to maintain adequate nutrition and hydration. Standard pain management regimens in HNC patients are generally reactive and include opioids in combination with various adjunct medications [4]. Mucositis-associated pain is notoriously opioid resistant; furthermore, opioids produce unwanted side effects including nausea, constipation, and central nervous system symptoms that may interfere with cancer treatment [5] and can lead to dependence [6, 7]. Therefore, it is imperative that analgesic adjuncts that may reduce the amount of opioids needed during HNC therapy be thoroughly evaluated. One promising analgesic adjunct beginning to be used in this patient population is gabapentin.

Gabapentin is a potent anti-inflammatory and neuromodulator agent that is frequently used in the treatment of neuropathic pain [8]. It has been studied in the context of HNC therapy as an adjunct for the management of acute pain, prevention (prophylaxis) of pain and treatment-related toxicity, and prevention of adverse events such as feeding tube placement. The primary purpose of this review is to summarize the available data on the use of prophylactic gabapentin in HNC patients undergoing concurrent chemoradiation. Prophylactic therapy is defined as the use of gabapentin before or at the start of radiation-based therapy regardless of whether the patient does or does not have pain. Given the sparsity of data on the subject, in order to provide the most clinically actionable data, this review summarizes data from randomized trials, prospective studies with historical controls, and retrospective studies. It is noteworthy that the randomized trials on this topic are all relatively small and thus gain only partial protection from unmeasured confounding based on their size.

## Methods

Searches were conducted on Pubmed, Web of Science, and Google Scholar to identify candidate manuscripts. Search terms included [gabapentin OR Neurontin] And [head and neck OR oral OR oropharyngeal] AND cancer. Once a study was accepted for inclusion in the review, its references were also searched for additional studies that may have been omitted. Two reviewers screened each article for eligibility, and data abstraction was conducted in duplicate. Discrepancies were resolved by group consensus. In order to be considered for inclusion, a study had to be an investigation of prophylactic gabapentin during concurrent chemoradiation for head and neck cancer. To be included, the study also needed to report one of the following outcomes: pain score, opioid use, feeding tube placement, or weight loss. Articles were excluded if they were (1) not published in the previous 10 years, (2) were case studies or case series, (3) were studies in which gabapentin was not administered prophylactically during chemoradiation for head and neck cancer, or (4) were studies in which the reviewers were unable to ascertain study characteristics or outcomes. A risk of bias assessment was conducted using Cochrane’s ROBINS-I tool. Assessments were conducted in duplicate and discrepancies were resolved by consensus.

A synthesis of results was conducted for outcomes for which comparable data could be identified. Critical outcome parameters consistently reported by multiple studies included reactive feeding tube placement and percent change from baseline weight. The number of feeding tubes placed was analyzed via a random effects model for the risk difference between controls and those receiving prophylactic gabapentin. The manuscripts that investigated weight loss presented point estimates, but no uniform measure of dispersion was present amongst the studies, so the *p*-values from each of the three studies with assessable risk of bias were combined using Fisher’s Method. In the analysis of feeding tube placement, for which traditional meta-analysis was possible, a sensitivity analysis was conducted by excluding one study which induced extreme heterogeneity into the result demonstrated by funnel plot assessment and I^2^. Unfortunately, the pain was not consistently reported among the identified studies. Each identified study that examined pain as an outcome utilized a different patient-reported outcome (PRO)-based pain measure tool, making synthesis impossible.

## Results

### Included studies

**Fig. 1:**
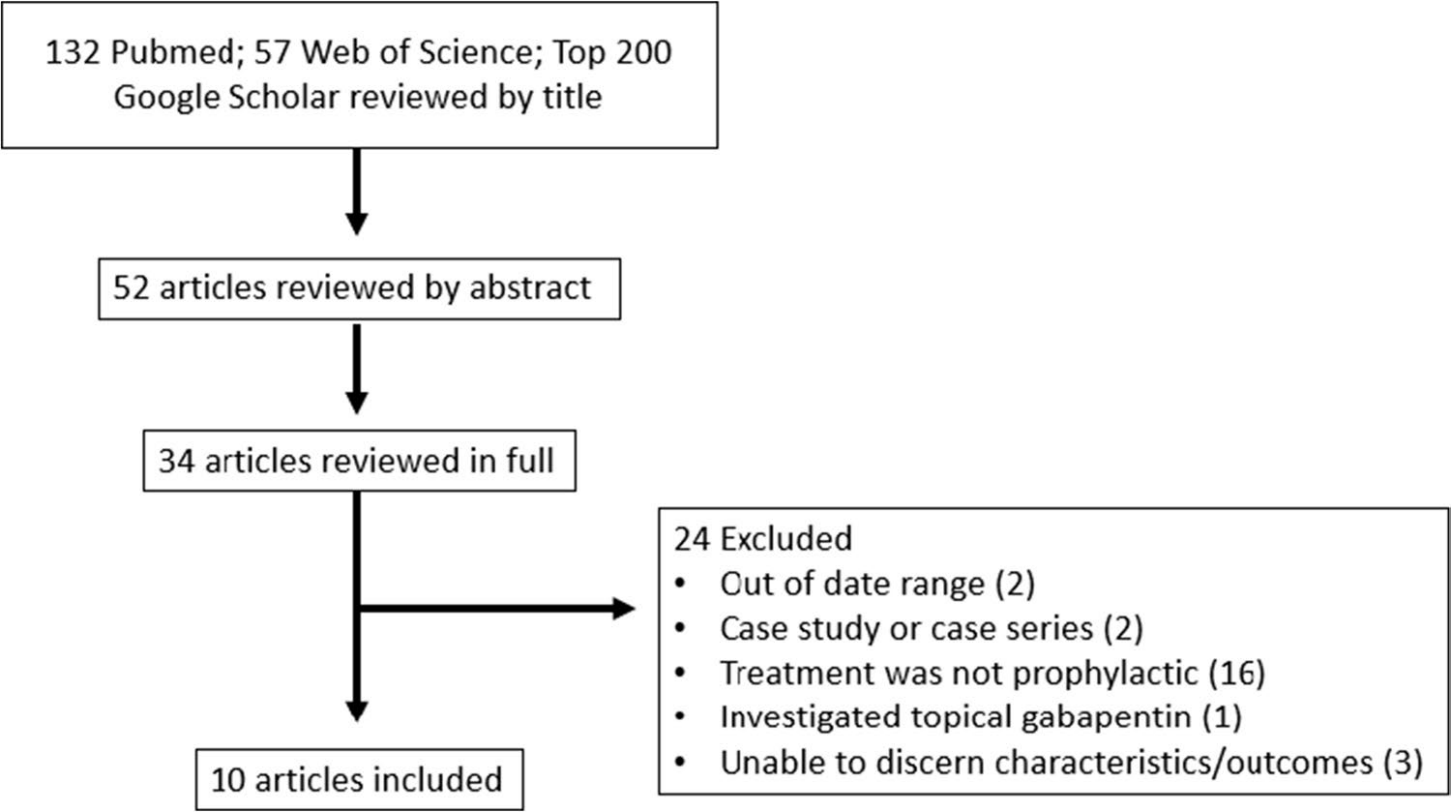
PRISMA diagram demonstrating inclusion and exclusion of studies

**Fig. 2:**
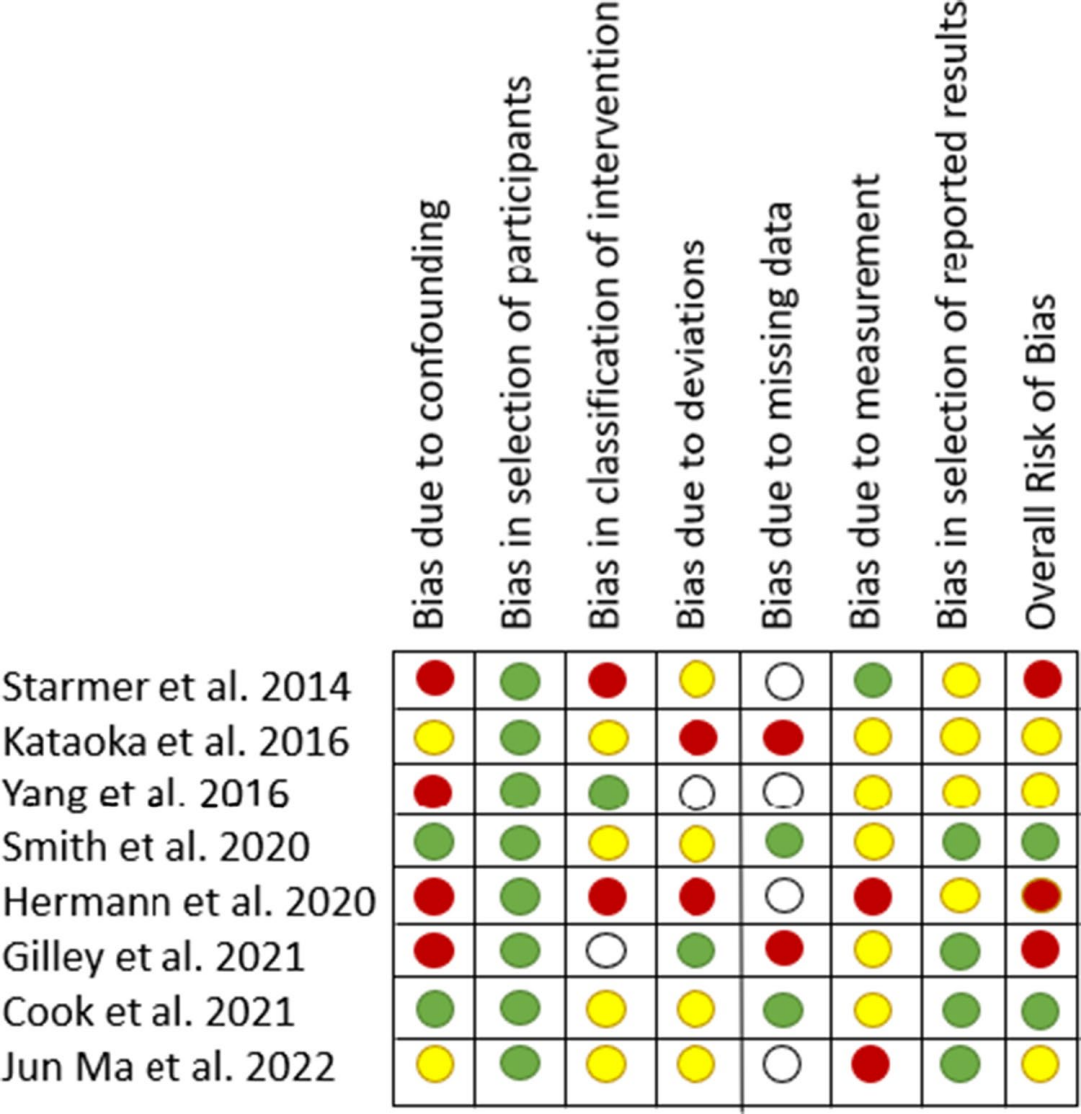
Risk of bias assessment for included studies

The results of the search are detailed in Fig. 1. A total of 132 search results were obtained on Pubmed and 57 additional articles were obtained through the Web of Science for all three search queries. These were reviewed for eligibility along with the top 200 Google Scholar results for each search query. From these articles, 52 article abstracts were reviewed and 34 articles were further reviewed in full. All references in these 32 articles were also reviewed by abstract for eligibility. Ten articles were determined to be eligible for inclusion in the meta-analysis, while the remaining articles were excluded for one or more of the following reasons: published more than 10 years before this review (2 studies), presenting a case study or series (2), gabapentin treatment was not administered prophylactically (16), gabapentin was administered topically instead of orally (1), and for difficulty discerning important study characteristics related to the outcome of interest for this meta-analysis (3). Details of the 10 included studies are presented in Table 1. Risks of bias associated with each included study were independently assessed by two reviewers and the consensus of these assessments is presented in Fig. 2.

**Table 1:** Details of the 10 included studies

Author	Study type	Year	Study population	Treatment administered	Pain scale	Conclusions
Starmer et al	Observational	2014	23 patients were treated with prophylactic gabapentin and compared with 23 matched historical controls	Gabapentin 2700 mg/day	4-point Likert	Maximum pain reduced in treatment arm (*p* = 0.0003) and shorter pain duration (*p* = 0.038). Patients in the treatment arm used fewer opioids but no statistical comparison was done. Prophylactic PEG tubes, but patients in the treatment arm began using them later (*p* = 0.013) and had them removed earlier (*p* = 0.029). Patients in the treatment group also lost less weight (*p* = 0.037)
Quon et al	Observational	2014	521 patient retrospective study; distribution of participants between arms unclear	Gabapentin unclear dosage	None	Significant difference in weight loss in favor of gabapentin (*p* = 0.0285)
Dong et al	Observational	2016	Retrospective study of 64 patients (31 treatment; 33 control)	Gabapentin unclear dosage	None	Significant difference in weight loss (*p* = 0.004)
Kataoka et al	RCT	2016	22 patients randomized to standard management + / − gabapentin	Gabapentin 900 mg/day	Visual Analog Scale 0–10 of average 24 h pain	Inconclusive results on pain, weight loss, feeding tube use, and quality of life
Yang	Observational	2016	192 patients identified by retrospective chart review (89 gaba; 103 non-gaba)	Gabapentin 2700 mg/day	None	Near significant difference in PEG tube placement (50/89 treatment vs. 71/103 control), and significant difference in PEG use (33/50 treatment vs. 64/71 control)
Smith et al	RCT	2020	79 patients randomized to standard management + / − gabapentin (41 gaba; 39 non-gaba)	Gabapentin — titrated between 300 and 2700 mg/day	Vanderbilt Head and Neck Symptom Survey pain subscale (0–10)	Statistically significant difference in pain favoring treatment group. No difference in PEG tube placement (15/41 treatment vs. 16/39 control). Marginally significant difference in weight loss (2.1% treatment vs. 8.1% control, *p* = 0.057)
Hermann et al	RCT without usual care control	2020	60 patients randomized to gabapentin vs. gabapentin + methadone	Gabapentin 2700 mg/day	EORTC Pain Scale (0–100)	Inconclusive results for gaba and gaba + methadone in pain. Only 2/29 patients in the Gaba arm received a feeding tube
Gilley et al	Observational	2021	185 patients (53 gaba; 132 historical controls)	Gabapentin 2700 mg/day	None	Gabapentin was associated with less chronic opioid use at 3mo post treatment (*p* = 0.021). PEG tubes less necessary in the treatment arm (18/53 vs. 82/132)
Cook et al	RCT	2021	58 patients (29 gaba; 29 standard management)	Gabapentin 1800 mg/day	Patient Reported Oral Mucositis Symptom item 1 score (0–100)	Inconclusive result on pain (*p* = 0.011), average opioid use (*p* = 0.32), weight loss (*p* = 0.81), but showed significantly higher PEG tube placement in the treatment arm (18/29 gaba vs. 6/29 control)
Jun Ma et al	Secondary analysis of two clinical trials	2022	92 patients (32 gaba 3600 mg; 31 gaba 2700 mg; 29 gaba 900 mg)	Gabapentin 900, 2700, 3600 mg	None	Proportion requiring opioids was reduced (37.5% 3600 mg; 61.3% 2700 mg; 93.1% 900 mg; *p* < 0.001). Feeding tubes were placed in each cohort (3/32 3600 mg; 14/31 2700 mg; 6/29 900 mg)

### Prophylactic gabapentin for pain control during chemoradiation for HNC

Gabapentin’s efficacy in the treatment of neuropathic pain makes it a plausible candidate for managing pain secondary to radiation treatment, which can be the result of radiation-induced inflammation, mucositis, or direct nerve injury. The first study meeting the inclusion criteria to investigate the use of prophylactic gabapentin for pain during head and neck cancer therapy was Starmer et al. in 2014 [9]. In this study, 23 HNC patients who received 2700 mg/day of prophylactic gabapentin were compared to historical controls who received standard pain management. The pain was measured on a 4-point Likert scale consisting of absent; mild; moderate; and severe pain. The duration of pain was recorded in days and the maximum pain score over the course of treatment was determined. Gabapentin treatment resulted in statistically significant reductions in both duration of pain and maximum pain score during treatment.

**Fig. 3:**
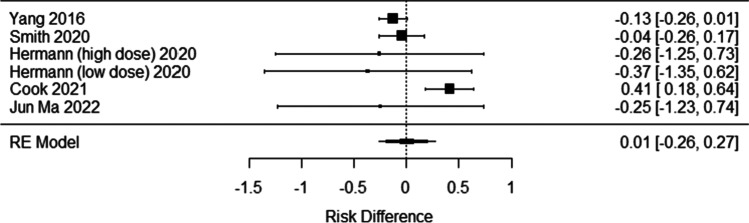
Forest plot examining the risk of feeding tube placement for patients on gabapentin versus control

In addition to Starmer, three randomized studies exist which investigated the impact of prophylactic gabapentin on pain during HNC treatment [10–12]. Unfortunately, each of the trials is small, and therefore, they receive limited benefit from randomization and have low power to detect mild to moderately sized treatment effects. In addition, given the frequency and oft profundity of drowsiness associated with substantial doses of gabapentin, even in studies that attempted blinding it is very likely that both patients and providers were aware of their likely treatment assignment. The first randomized trial of gabapentin in this context was performed by Kataoka et al. in 2016 [11], which randomized 22 HNC patients to standard pain management with or without gabapentin at 2700 mg/day. The authors recorded the maximum visual analog score (VAS) over the course of treatment and compare the median maximum scores between the two treatment groups. No difference in VAS median maximum scores was detected. However, given the size of this trial, the statistical analyses likely suffered from a severe lack of statistical power. Indeed, the manuscript contains no calculation or discussion of the trial’s statistical power. Figure 3 of the Kataoka et al. manuscript displays the distribution of VAS scores from the trial. Close examination of this figure suggests that the VAS scores from the two treatment groups may have differed, just not in the median value. Although the median VAS scores are comparable between the two treatment groups throughout the course of treatment, the distance from the median to the 75th percentile is notably contracted in the gabapentin arm suggesting that those in the upper portion of the pain scale may have received some benefit from the addition of gabapentin to their pain regimen. To further assess the data presented by Kataoka et al., we used a plot digitizer to extract the 25th, 50th, and 75th quartiles from Fig. 3. The interquartile distance between the 75th and 50th quartiles was then normalized by the distance between the 50th and 25th percentiles to account for the changing variability in the distribution of pain scores over the course of treatment in their trial. We then performed a Wilcoxon rank sum test which demonstrated a statistically significant reduction in pain favoring the gabapentin group among the higher pain scores (*p* = 0.026). Though the post hoc nature of this analysis does not conclusively prove the assertion, it is suggestive that if Kataoka et al. had undertaken a longitudinal quantile regression of the 75th percentile, they may have identified a difference in pain scores between their treatment groups.

The second randomized trial of prophylactic gabapentin during HNC chemoradiation treatment was performed by Smith et al. The planned recruitment for this randomized trial was 150 patients with an interim analysis at 75 patients. Interim analysis at 79 patients demonstrated efficacy for both primary and secondary endpoints and the trial was halted [10]. HNC patients in this trial were randomized to standard pain management with or without gabapentin. Patients randomized to gabapentin were started on 100 mg/day on day 1 of radiation-based therapy with a dose titrated upwards weekly as tolerated. The most common gabapentin dose over the trial period was 900 mg/day in the gabapentin treatment group. In this trial, the pain was measured using the Vanderbilt Head and Neck Symptom Survey’s pain subscale, a combination of 4 questions related to pain severity individually reported on a 0–10 scale. These investigators identified a difference in pain scores in favor of gabapentin usage (OR = 0.549, 95% CI: (0.364, 0.827), *p* = 0.004). In the Smith et al. manuscript, a similar boxplot was presented to that in Kataoka et al. [11] showing similar reductions in the 75th percentile of pain scores with gabapentin treatment. In contrast to Kataoka et al., however, Smith et al. also demonstrated significant reductions in the median pain score in HNC patients treated with gabapentin.

Similar to Kataoka et al., Smith et al. suggested that gabapentin affects those at high levels of pain more profoundly than patients at lower pain levels. The differential effect of gabapentin depending on pain score level implies the effect of gabapentin will differ over the course of therapy, since pain scores tend to be low at the beginning of HNC treatment and increase over time. This suggests that statistical analyses relying on differences in mean pain scores will lack power, even beyond what is estimated based on a study’s design. This is due to the fact that comparisons of mean pain scores are pooling a heterogeneous treatment effect between patients with either no or mild pain, who receive little benefit, and patients with moderate to severe pain who receive greater benefit from gabapentin treatment.

The third trial of prophylactic gabapentin for pain control during chemoradiation for HNC, published by Cook et al., randomized 58 patients to receive standard management with or without 2700 mg/day of gabapentin [12]. As the only trial that was double-blind, this study had the least risk of bias by design, although the effectiveness of blinding 2700 mg/day of gabapentin is questionable. Pain was measured using a single item from the patient-reported oral mucositis symptom scale ranging from 0 to 100. Figure 2a of this manuscript provides the mean pain score over time during the trial, and contrary to what the previous studies show, it demonstrates that there was a small baseline imbalance in favor of the control arm which persisted throughout the course of therapy without any apparent effect of gabapentin on the mean value (*p* = 0.11). This baseline imbalance would have been even more severe if not for one patient in the control group with an extremely high baseline pain score. Though this study benefits from its double-blind design, like Kataoka et al., it lacks statistical power to detect moderately sized effects of gabapentin both due to its small size and its choice of mean-based analysis. This study does provide some degree of evidence against very large effects which it had modest power to detect. It is noteworthy that this study had a large number of patients at baseline with elevated pain scores prior to therapy and a greater proportion of these patients being assigned to the gabapentin arm. It is possible that pre-HNC treatment pain is less likely to be neuropathic in nature and therefore less responsive to the gabapentin therapy.

Due to the variety of instruments selected by the investigators in these studies to quantify pain, it would be difficult to perform a meaningful meta-analysis of this data. The current state of evidence rests on the fact that there are two clinical trials demonstrating improved pain with gabapentin during HNC treatment and two trials which are inconclusive due to their profound lack of statistical power to detect moderate improvements in pain scores. Estimates using empirical data suggest that the Cook et al. study had an estimated statistical power of just 25.5% [13]. At less than half the size, the Kataoka study’s statistical power was certainly negligible.

### Prophylactic gabapentin to reduce opioid use during chemoradiation for HNC

Several studies have examined whether prophylactic gabapentin during HNC chemoradiation therapy can either eliminate, reduce the dosage, or reduce the timeframe in which opioid therapy is necessary. The first study to examine this outcome was by Stramer et al. in 2014. They observed reduced doses of opioids among patients given gabapentin during HNC therapy compared to historic controls, but no statistical comparisons were performed to support these observations [9]. In contrast, the randomized trial by Cook et al. found no significant difference in opioid dose between treatment groups (*p* = 0.32) [12]. In a comparative-effectiveness study utilizing data from two clinical trials, Jun Ma et al. demonstrated a dose-dependent effect of gabapentin reducing the need for opioids for pain management from 93.1% at 900 mg/day of gabapentin to 37.5% at 3600 mg/day of gabapentin (*p* < 0.001) [14]. In addition, Gilley et al. demonstrated that patients on prophylactic gabapentin during therapy have less persistent opioid use at 3 months post-radiotherapy [15].

Overall, the majority of studies examining the effect of gabapentin treatment on opioid use during HNC therapy have demonstrated an association between gabapentin use and a reduced need for opioids, supporting the hypothesis that gabapentin reduces opioid use in HNC patients undergoing chemoradiation.

### Prophylactic gabapentin to reduce feeding tube placement during chemoradiation for HNC

A number of studies have examined the effect of prophylactic gabapentin on swallow function and the need to provide enteral nutrition through feeding tubes. Starmer et al. demonstrated that gabapentin use was associated with patients beginning the use of their prophylactically placed feeding tubes later into HNC therapy (*p* = 0.013) and was associated with earlier feeding tube removal (*p* = 0.029) compared to historic controls who received standard management [9]. One year follow-up demonstrated healthy swallow function in the patients who received gabapentin during HNC treatment, although this follow-up study lacked any comparator group [16]. In a retrospective study by Yang et al., fewer feeding tubes were placed in patients receiving gabapentin during radiation therapy for HNC, but the finding was not statistical significance (50/89 gabapentin vs. 71/103 non-gabapentin, *p* = 0.094) [17]. In contrast, Yang et al. found that 33 of the 97 patients who utilized PEG tubes were taking gabapentin, while 17 of the 24 patients who did not utilize their PEG tube were taking gabapentin (*p* < 0.01), and demonstrated that gabapentin use was independently associated with a lower risk of PEG tube utilization (*p* < 0.01). The randomized trial of prophylactic gabapentin vs. standard management by Smith et al. found no difference in feeding tube placement rate between the two treatment groups (15/41 gabapentin treatment vs. 16/39 standard management, *p* = 0.858) [10]. In Hermann et al., 9/29 patients on high-dose gabapentin had a feeding tube placed during HNC treatment while only 4/29 patients on low-dose gabapentin underwent feeding tube placement during HNC treatment [18]. In Jun Ma et al. dose–response study with gabapentin, there was no observed increase in feeding tube placement with increasing gabapentin dose. Indeed, the group of patients who received the middle dose of gabapentin, 2700 mg/day, required the largest proportion of feeding tubes among the three (3/32 3600 mg/day; 14/31 2700 mg/day; 6/29 900 mg/day) [14]. In contrast, Cook et al., in their randomized, double-blind trial, found that the gabapentin treatment group required more feeding tubes than the control group (18/29 gabapentin vs. 6/29 control, *p* = 0.001) [12].

**Fig. 4:**
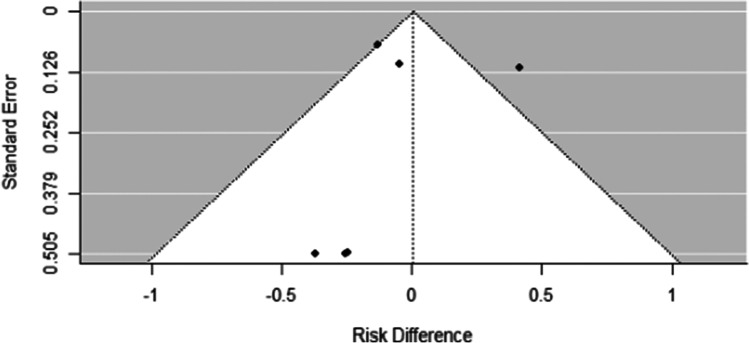
Funnel plot for the analysis of feeding tube placement, noting the anomalous finding in Cook et al

Given the risk of an invasive procedure and the emotional distress feeding tube placement can induce, the finding by Cook et al. must be seriously considered. Unlike most of the outcomes discussed in this review which are measured using varying scales, feeding tube placement is consistently reported and is therefore a candidate for meta-analysis. Data from five studies were pooled using a random-effects model (see Fig. 3). There was substantial evidence of heterogeneity between studies (*I*^2^ = 72.1%, *Q* = 16.93, *p* = 0.005, Fig. 4), which arises specifically from the Cook et al. data [12]. The pooled risk difference suggests there is no evidence for difference between the groups (RD = 0.64%, 95%CI: (− 25.8%, 27.1%), *p* = 0.962). Sensitivity analysis, which eliminated the Cook et al. study, removed the heterogeneity (*I*^2^ = 0.0%, *Q* = 0.84, *p* = 0.932) and demonstrated a pooled risk difference that nearly missed statistical significance in favor of gabapentin (RD =  − 11.1%, 95%CI: (− 22.3%, 0.2%), *p* = 0.054). This meta-analysis suggests that despite the findings in Cook et al., there is no evidence for an increased need for feeding tube placement among those taking prophylactic gabapentin, and the degree of heterogeneity between the Cook et al. study and others suggests some other undefined process may have influenced the data from that study.

### Prophylactic gabapentin to reduce weight loss during chemoradiation for HNC

Difficulty maintaining weight during cancer therapy can be secondary to a number of complications including depression, loss of appetite, pain, mucositis, and dysphagia. Starmer et al. found that patients on gabapentin lost less weight than controls (7.45% treatment vs. 11.0% control, *p* = 0.037) [9]. Quon et al. and Dong et al., both retrospective studies, claimed a significant reduction in weight loss in patients taking gabapentin during HNC treatment compared to patients not taking gabapentin, but did not provide sufficient information to allow an assessment of the risk of bias. A difference that neared statistical significance was also present in Smith et al. (2.1% treatment vs. 8.1% control, *p* = 0.057) [10]. Cook et al. found no significant difference in weight loss (11.4% treatment vs. 10.7% control, *p* = 0.81) [12]. Unfortunately, due to the fact that no measures of dispersion (SD or IQR) are present in Starmer et al., the usual random effects model for meta-analysis is not employable. Alternatively, by employing Fisher’s method, a pooled *p*-value from the three studies (Starmer et al.; Smith et al.; Cook et al.) can be obtained and gives an overall *p* = 0.047 in favor of gabapentin reducing weight loss during chemoradiation therapy for HNC.

## Discussion

Currently, few studies of prophylactic gabapentin use in HNC patients during concurrent chemotherapy and radiation have been completed. Among completed studies, few are randomized trials, most are very small, and all but two have a moderate to high risk of bias. Available evidence from these studies supports the use of prophylactic gabapentin in HNC patients to prevent treatment-induced pain, with the most likely benefit in patients who develop moderate to severe HNC treatment-related pain. Data also support the use of prophylactic gabapentin as a means of reducing opioid use during HNC treatment.

One somewhat concerning finding in the Cook et al. study was that their treatment group required the placement of substantially more feeding tubes than the control group. However, the meta-analysis of current studies demonstrates that there is no evidence for increased feeding tube placement in HNC patients who receive gabapentin during treatment. Indeed, if the results of Cook et al. are excluded due to high heterogeneity between this trial and other published studies, there is a trend toward a reduced risk of feeding tube placement with gabapentin use during HNC treatment. Although the Cook finding is somewhat concerning in isolation, when viewed in the totality of the evidence, it appears to be anomalous.

Furthermore, by employing Fisher’s method, we were able to identify that prophylactic gabapentin use is associated with less weight loss during HNC treatment. Therefore, overall, current data support the use of prophylactic gabapentin in HNC patients to reduce pain, opioid use, and weight loss during treatment. However, caution should be used when applying these findings to patient care due to the small sizes of most currently available studies for meta-analysis. A large, multicenter randomized trial of prophylactic gabapentin use in HNC patients during concurrent chemoradiation therapy, which is adequately powered to detect clinically meaningful changes in pain scores, duration of pain, opioid use, and weight loss, is needed.

## Data Availability

The data used in this study is publicly available through the referenced articles.
